# Gestational Exposure to a Viral Mimetic Poly(I:C) Results in Long-Lasting Changes in Mitochondrial Function by Leucocytes in the Adult Offspring

**DOI:** 10.1155/2013/609602

**Published:** 2013-09-19

**Authors:** Cecilia Giulivi, Eleonora Napoli, Jared Schwartzer, Milo Careaga, Paul Ashwood

**Affiliations:** ^1^Department of Molecular Biosciences, School of Veterinary Medicine, University of California, Davis, CA 95616, USA; ^2^Medical Investigations of Neurodevelopmental Disorders (MIND) Institute, University of California, Davis, CA 95616, USA; ^3^Department of Medical Microbiology and Immunology, School of Medicine, University of California, Davis, CA 95616, USA

## Abstract

Maternal immune activation (MIA) is a potential risk factor for autism spectrum disorder (ASD) and schizophrenia (SZ). In rodents, MIA results in changes in cytokine profiles and abnormal behaviors in the offspring that model these neuropsychiatric conditions. Given the central role that mitochondria have in immunity and other metabolic pathways, we hypothesized that MIA will result in a fetal imprinting that leads to postnatal deficits in the bioenergetics of immune cells. To this end, splenocytes from adult offspring exposed gestationally to the viral mimic poly(I:C) were evaluated for mitochondrial outcomes. A significant decrease in mitochondrial ATP production was observed in poly(I:C)-treated mice (45% of controls) mainly attributed to a lower complex I activity. No differences were observed between the two groups in the coupling of electron transport to ATP synthesis, or the oxygen uptake under uncoupling conditions. Concanavalin A- (ConA-) stimulated splenocytes from poly(I:C) animals showed no statistically significant changes in cytokine levels compared to controls. The present study reports for the first time that MIA activation by poly(I:C) at early gestation, which can lead to behavioral impairments in the offspring similar to SZ and ASD, leads to long-lasting effects in the bioenergetics of splenocytes of adult offspring.

## 1. Introduction

The most recent estimates indicate that the prevalence of autism spectrum disorders (ASD) in the United States has raised to 1 in 54 boys and 1 in 252 girls [1]. Although increased awareness and changes in diagnostic criteria have been proposed as the major contributors to this increased prevalence [2], as of today, the etiopathology of disorders like ASD and schizophrenia (SZ) remains largely unknown.

Several studies have suggested that impaired mitochondrial function and altered energy metabolism in individuals with ASD may contribute to their social and cognitive deficits [3–5], and recent reports indicate the presence of mitochondrial dysfunction (MD) in brain, skeletal muscle, and peripheral blood mononuclear cells (PBMC) from children with ASD. The MD in ASD is generally characterized by lower complex I activity accompanied, in a subset of cases, by deficits in other complexes [6–8]. 

Beside their critical role in a number of pathways, spanning from ATP production (via oxidative phosphorylation), one-carbon metabolism regulation, heme biosynthesis, fatty acid catabolism, and branched chain amino acid metabolism [9], mitochondria may also impact the immune response and vice versa [10–12]. For example, human neutrophil mitochondria are involved in several functions such as chemotaxis, respiratory burst activity, maintenance of cell shape, and apoptosis [13–17]. Furthermore, neutrophil phagocytosis may involve the incorporation of some mitochondrial proteins into the phagosome [18]. In addition, mitochondria can be involved in the immune response by providing part of the metabolic pathway for Gln, in a process named “glutaminolysis” [19, 20]. Interestingly, Gln is implicated in the expression of  NADPH oxidase components, cytokine production in lymphocytes, and macrophage, and as a provider for substrates required for nucleic acid synthesis [21–25]. Taken together, these lines of evidence unveil a link between mitochondria and immune response [10–12]. Indeed, deficits in bioenergetics have been reported in lymphocytes from children with ASD enrolled in the case-control population-based Childhood Autism Risk Genetics and Environment (CHARGE) Study [26, 27]. Children with ASD in this study display a number of immune dysfunctions including abnormalities in monocytes, T cells and NK cell responses [28]. These observations suggest the presence of a genetic background that results in a distinct immune profile in responses to a variety of triggers, among them psychological stressors, exposure to chemical triggers, and infectious agents [29, 30]. 

Considering that (i) mitochondria are inherited maternally via oocyte, (ii) maternal diet or immune activation during pregnancy has an impact on fetal metabolic and immune programming [31–33], and (iii) offspring born to pregnant mice injected with poly(inosinic:polycytidylic acid) (poly(I:C)), a synthetic double-stranded RNA that mimics viral infection via activation of Toll-like receptor-3 (TLR3), at embryonic day 12.5 (E12.5), display core behavioral symptoms of ASD [34, 35] and SZ [34], it is hypothesized that prenatal exposure of mothers to an immunogenic response, that is, poly(I:C) elicits changes in mitochondrial function in splenocytes from progeny lasting into adulthood. Exposure to TLR ligands can lead to maternal hypertension, vascular dysfunction, and proteinuria in pregnant animals but not in nonpregnant animals [36–38] suggesting the occurrence of a differential immune response/pathway during pregnancy. Differences are also evident between pregnant individuals with human placentas and patients with preeclampsia showing greater expression of TLR3, along with TLR2, TLR4, and TLR9, compared to nonpreeclampsia mothers [39, 40]. These data suggest that TLR signaling may be involved in placental deficiencies/abnormalities that may provide a framework for altered fetal programming. Of note, trophoblastic inclusions, which are also observed in preeclampsia and other placental defects, were reported to be increased in placenta from mothers of children with ASD compared to controls [41]. Furthermore, maternal exposure to various pathogens, including viruses, significantly increases the risk for ASD and SZ [42–48]. Considering that maternal exposure to various pathogens is associated with ASD and SZ, the critical link between prenatal maternal infection and postnatal brain and behavioral pathology seem to be the maternal immune response, including cytokine production [47, 49–54], which may contribute to the fetal imprinting of the neuroimmune response and, possibly, mitochondria-mediated metabolic responses. Although it is already known that upon poly(I:C) injection, the induction of maternal cytokines alters the expression of several cytokines in the fetal brain (with only IL-1*β* remaining elevated at 24 h [53] with only a few changes during adulthood), it is unknown if maternal immune activation (MIA) also causes chronic changes in the bioenergetics of immune cells (such as splenocytes) of adult offspring. 

In this study, we sought to determine whether MIA in pregnant dams alters mitochondrial function in splenocytes from affected offspring. To test this, dams were exposed to poly(I:C) on gestational day 12.5 to induce MIA. This stage of gestation correlates with the late first trimester in humans [55], coincidental with the time that infections are most closely linked to increased incidence of ASD and SZ [47, 48]. The present study reports for the first time that MIA activation by poly(I:C) at early gestation, which can lead to impairments in multiple psychological domains, is associated with mitochondrial changes in immune the cells of adult offspring.

## 2. Materials and Methods

### 2.1. Animals

Male and female C57BL/6J (Jackson Laboratory, Sacramento, CA, USA) mice were bred and maintained by the Center for Laboratory Animal Research, at University of California, Davis, and maintained at ambient room temperature on a 12 h light/dark cycle (lights on at 06:00 h). Food and water were provided *ad libitum*. All procedures were performed with approval by the Institutional Animal Care and Use Committee, University of  California, Davis, and in accordance with the guidelines provided by the National Institutes of Health Guide for the Care and Use of Laboratory Animals.

### 2.2. Treatment and Behavioral Assessment

Mice were mated overnight and females were checked daily for the presence of seminal plugs, noted on gestational day 0.5 (G0.5). On G12.5, pregnant female mice were weighed and injected with a single dose (20 mg/kg; i.p.) of poly(I:C) (Sigma Aldrich, St Louis, MO, USA) or saline vehicle (SHAM) as previously described [35]. Each dam was returned to its cage and left undisturbed until the birth of its litter. All mice pups remained with the mother until weaning on postnatal day 21, at which time mice were group-housed 3-4 per cage with same-sex littermates. Mice born from poly(I:C)-treated dams exhibited autism-like behavioral deficits including reduced social approach, increased ultrasonic vocalizations, and repetitive marble burying behaviors [35].

### 2.3. Splenocyte Isolation

One week following behavioral testing, 12 wk old mice were sacrificed by cervical dislocation and spleens were collected for tissue processing. While spleen is constituted by a variety of cells relevant to the immune response (including T- and B-lymphocytes, dendritic cells, and macrophages), it has recently been shown that spleens from the offspring of MIA mice elicited by poly(I:C) provide a more homogeneous preparation enriched in granulocytes compared to the preparation obtained from whole blood [56]. Briefly, spleens were homogenized into single cell suspensions by gently pushing them through a 100 *μ*m nylon mesh filter (Fisher Sci) into PBS at 4°C. Cells were then pelleted, and RBCs were lysed using ACK lysis buffer according to the manufacturer's instructions (Gibco). Cell suspensions were kept on ice until analyzed for mitochondrial activity. Cell viability was determined by trypan blue staining and found to be about 90%. 

### 2.4. Mitochondrial Activities

The oxygen uptake of  intact cell suspensions (10^6^ cells/mL) obtained as described above was measured by using a Clark-type O_2_ electrode from Hansatech (King's Lynn, UK) at 22°C. Cells were incubated in the presence of 5 mM glucose in calcium and magnesium-supplemented HBSS buffer without phenol red at 20–22°C. NADH, succinate, and cytochrome oxidase activities were evaluated under phosphorylating conditions as described before [6, 30]. To this end, cells were permeabilized with a controlled treatment with digitonin [57] by adding 60 *μ*g/mL 2x recrystallized digitonin for 2 min. The solubilization was stopped by the addition of 1 mg/mL BSA. Oxygen consumption rates were evaluated in the presence of 1 mM ADP plus 1 mM malate-10 mM glutamate followed by the addition of 5 *μ*M rotenone; 10 mM succinate followed by the addition of 3.6 *μ*M antimycin A, and 10 mM ascorbate and 0.2 mM *N,N,N*′*,N*′*-*tetramethyl-*p-*phenylenediamine followed by the addition of 1 mM KCN.

### 2.5. Statistical Analyses

All mitochondrial experiments were run in triplicates. Mitochondrial data were expressed as mean ± standard error. Student's two-tailed *t*-test was used to evaluate the differences between offspring of poly(I:C)-treated and SHAM-treated dams. 

## 3. Results and Discussion

### 3.1. Deficits in Complex I in Splenocytes from Mice Gestationally Exposed to Poly(I:C)

**Figure 1:**
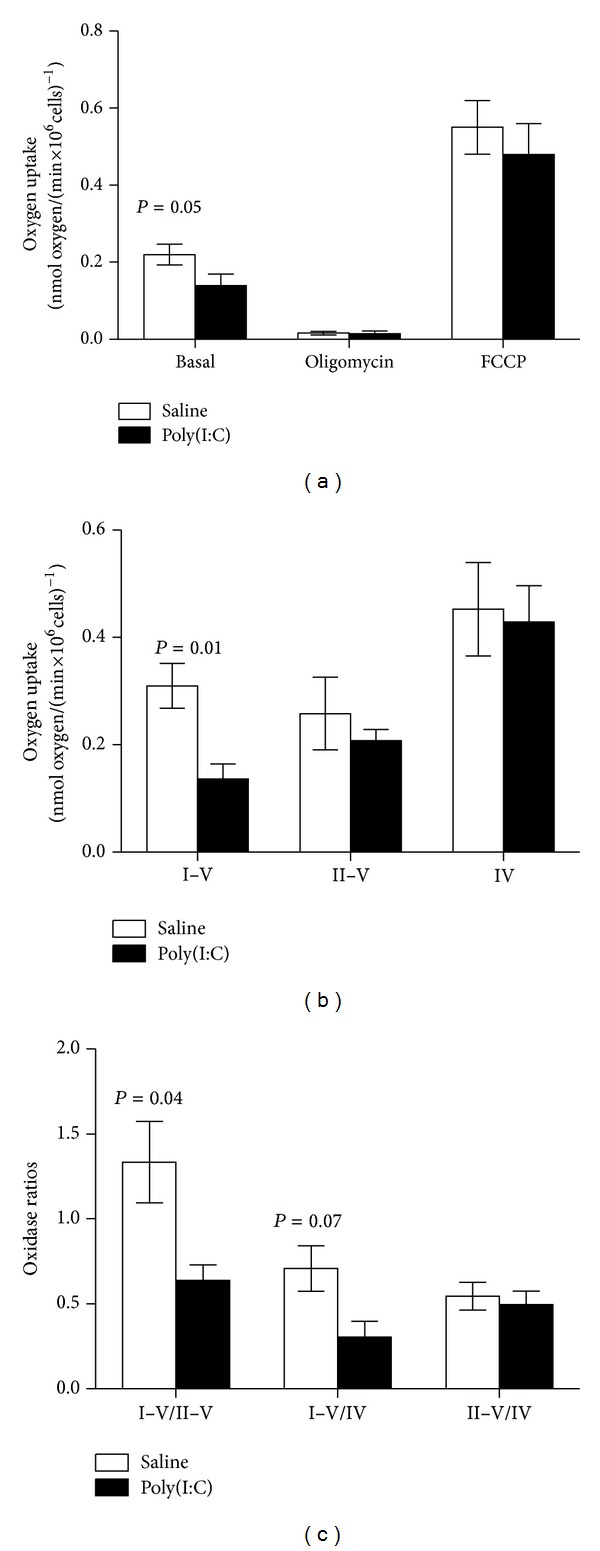
Mitochondrial outcomes in splenocytes. Splenocytes from offspring (10–12 weeks old) poly(I:C)-(*n* = 8) or saline-(*n* = 8) treated dams at 12.5 gestational day were isolated, and mitochondrial outcomes were tested as described in detail in Section 2. (a) Oxygen uptake of intact cells was determined using a Clark-type electrode in Hanks balanced salt solution (HBSS) supplemented with 6 mM glucose as substrate. Basal (only glucose), oligomycin (0.2 *μ*M), and FCCP (20 *μ*M) were added, and the initial rates of oxygen uptake were calculated and normalized by million cells. (b) Permeabilized cells were tested for their capability to consume oxygen coupled to ATP production by supplementing the media with malate-glutamate (or NADH oxidase comprised by complexes I–V), succinate with rotenone (or succinate oxidase comprised by complex II–V), and TMP-ascorbate for cytochrome *c* oxidase activity (or complex IV). (c) ratio of rates of oxygen consumption in the presence of various substrates derived from (b).

Splenocytes from adult mice born to either SHAM- or poly(I:C)-treated dams were isolated for mitochondrial function testing. Given that most of the oxygen uptake by cells is linked to ATP production via oxidative phosphorylation, this parameter was evaluated in intact cells in the presence of glucose (Figure 1(a)). The rate of oxygen uptake by intact cells from SHAM-treated dams was 0.22 ± 0.03 nmol oxygen × (min × 10^6^ cells)^−1^. Under the same conditions, this rate was decreased by 36% in poly(I:C)-treated dams (Figure 1(a)). Addition of oligomycin, an inhibitor of ATPase, was used to stop the fraction of oxygen utilized to synthesize ATP via mitochondria. In both groups, more than 90% of the total oxygen uptake was inhibited by oligomycin, supporting the previous assumption that most—if not all—oxygen uptake by these cells was derived from oxidative phosphorylation. The oxygen uptake resistant to oligomycin, considered somewhat equivalent to State 4 (nonphosphorylating mitochondria), was not different between groups. This result indicated that the proton leak across the inner mitochondrial membrane was similar in both groups, suggesting no major mitochondrial membrane damage by either treatment. Addition of FCCP, an uncoupler of electron transport and ATP synthesis, increased significantly the basal oxygen uptake to a similar extent in both groups (2.5- to 3-fold) with no changes between treatments, suggesting that the maximum respiratory capacity was similar in both groups. 

Coupling between oxygen uptake and ATP production was evaluated by the respiratory control ratio in intact cells (RCR). Mitochondria from either treatment showed a significant coupling with glucose as a substrate (with malate-glutamate, RCR = 3.5 ± 0.4 and 2.7 ± 0.3; with succinate, RCR = 4 ± 1 and 6.2 ± 0.5, for saline and poly(I:C), resp.) with no statistical differences between treatment groups. This result indicated that mitochondria were highly coupled and provided a means of support for their integrity during the testing process.

Phosphorylating mitochondria from splenocytes of SHAM animals in the presence of an NAD-linked substrate (such as malate) consumed oxygen at a rate of 0.31 ± 0.04 nmol oxygen × (min × 10^6^ cells)^−1^. Phosphorylating mitochondria from offspring of poly(I:C)-treated dams showed a significant decrease in oxygen consumption (by 55%; *P* < 0.01; Figure 1(b)). By adding rotenone, an inhibitor of Complex I, and succinate, a substrate for complex II, the segment comprising from complex II to complex V was evaluated. No differences in terms of oxygen uptake were observed between controls and poly(I:C) suggesting that the deficit in offspring of poly(I:C)-treated dams was located at the level of complex I. Confirming this result, complex IV activity was not different between treatments (Figure 1(b)) suggesting that mitochondrial mass was equivalent between groups. However, attempts to directly evaluate complex I activity were unsuccessful due to the limited amount of biological material. 

The ratios among complexes need to be preserved to provide suitable oxidation of substrates [58]. To this end, the ratios of electron transport chain activities indicated that both treatments allow oxidizing FAD-linked substrates (such as fatty acids) similarly, whereas a significantly lower oxidation of NAD-linked substrates (such as glucose) was evident in the poly(I:C)-treated condition compared to controls (Figure 1(c)). This imbalance in the complexes' ratios suggests that splenocytes from offspring of dams exposed to poly(I:C) use preferentially fatty acids over glucose as their main substrate for mitochondrial oxidative phosphorylation.

These results are consistent with the MD observed in lymphocytes from ASD children characterized by lower complex I activity and accompanied, in some cases, by deficits in other complexes and/or pyruvate dehydrogenase [6–8]. 

The cytokine production (IL-1*β*, IL-6, IL-10, IL-17, and TNF-*α*) from ConA-activated splenocytes obtained from adult offspring of poly(I:C)-treated animals was not different from that of SHAM-treated animals (see Supplementary Material available online on http://dx.doi.org/10.1155/2013/609602). This is consistent with the findings of others utilizing a similar MIA model in which only a handful of cytokines was still increased in early adulthood (frontal corteces IL-1*α*, IL-6, IL-10, and IL-9; cingulate corteces IL-10 and IFN-*γ*; none in hippocampus or serum [33]).

## 4. Conclusions

The aim of this study was to evaluate mitochondrial function in splenocytes from offspring gestationally exposed to an acute viral mimetic, that is, poly(I:C), to induce MIA. Our results indicate that the exposure of dams to a single dose of poly(I:C) at gestational day 12.5 likely triggers a TLR3-mediated response in the mother that is transmitted transplacentally to the offspring. In particular, the proinflammatory cytokine IL-6 has been proved to be a key intermediary in the behavioral changes observed in the offspring of dams treated with poly(I:C) [59]. Moreover, blocking IL-6 with antibodies prevents behavioral changes in the offspring [60], and poly(I:C)-induced MIA in IL-6 KO does not result in behavioral changes in the offspring [60]. These data seem to suggest a role for IL-6 in MIA-induced behavioral changes, although we cannot exclude other inflammatory agents such as type 1 interferons which have also been shown to take part in the response to poly(I:C) [61]. This MIA imprints a fetal programming that can still be detected during adulthood characterized by abnormal behaviors resembling those of ASD [34, 35] and SZ [34] and, biochemically, by a lower oxidative phosphorylation capacity in mitochondria within intact cells and isolated mitochondria. This suggests that prenatal immune changes ensuing the maternal poly(I:C) administration are likely to imprint the long-lasting changes in the bioenergetics of the adult offspring splenocytes. While, at the normal murine fetomaternal interface, immune cells such as neutrophils, macrophages, and NK cells are assumed to be excluded from the placenta and localized only in the decidua [62], treatment with poly(I:C) disrupts this normal distribution and induces a significant increase in the levels of proinflammatory cytokines in the placenta and a large migration of immune cells, primarily NK cells from the decidua towards the placenta, invading the spongiotrophoblast and then the labyrinth [63]. Trophoblasts, which express TLR3 [63], play a role in coordinating the maternal innate immune response to infection at the fetomaternal interface [62–64] and, especially in this case, in response to viral infection.

These results beget the question, what is the link between lower complex I activity (or lower oxidative phosphorylation) in the offspring and an acute maternal immune response? A growing body of evidence is placing mitochondria at the center of bioenergetics and immune response/inflammation. Immunity to infection is also dependent on mitochondria function by regulating the synthesis of both pro- and anti-inflammatory cytokines [65–69]. More recently, the view that mitochondria act as a platform facilitating innate immune responses adds to our understanding of the molecular complexity of sensor and adaptor interactions that promote effective host defense [11, 70]. 

Therefore, an emerging concept is that innate immune signaling is regulated by basic host metabolic functions. For instance, Toll-like receptor signaling activates mitochondrial biogenesis during critical illness [71–73], perhaps in response to increased oxidative damage in host cells [71, 74, 75]. Acute inflammation is accompanied by increases in inflammatory cytokines sustained by glycolysis, whereas chronic inflammation is sustained by less inflammatory cytokines, with more reparative features fueled mainly by mitochondria-derived ATP [76]. Thus, in this study, MIA seems to imprint the immune cells of adult offspring with this more glycolytic stage resembling the influence of an acute inflammation, without switching back to the less inflammatory response. Without pointing at cause or consequence, it is interesting to note that the changes in bioenergetics in the immune cells (and not their immune response or the immune response in brain or serum [33]) segregate with the abnormal behaviors observed in this MIA model [35]. However, a number of studies have shown differential MIA induction and behavioral responses depending on the gestational exposure stage [77–79]. This would suggest the existence of a window of vulnerability to infection during gestation for the onset of different behavioral defects, which may be reflected also on the mitochondrial function of the offspring. 

The above effects can be explained by (i) the transfer of immune cells and/or cytokines from mother to the fetus at the maternal-fetal interface and (ii) a genetic predisposition/susceptibility of the offspring that, in association with maternal viral or bacterial infections, might increase the risk of long-lasting behavioral and immune changes [35]. Furthermore, we cannot exclude the possibility that the relatively high doses of poly(I:C) used in this study could have affected the well-being of the mother and therefore that of the fetus. For instance, poly(I:C) inhibits the development of diabetes in the NOD mouse [80] whereas the development of diabetes in diabetes-prone BB rats is poly(I:C)-dose dependent [81–84]. Indeed, a recent report indicated that at least some nongenetic risk factors are shared between ASD and SZ, in particular, diabetes, exposure to drugs, nutritional deficiencies, and infectious agents among others [85]. 

Considering the mechanisms described above, studies are now needed to clearly identify the key players affected in this acute viral response in order to evaluate the increase in risk of either ASD or SZ that is associated with these (and other [30, 86, 87]) modifiable environmental factors to elicit public health interventions.

## Supplementary Material

Supplementary Methods
Detailed description of cell culture conditions, cytokine quantification, and statistical analysis used for the immune response-related data.
Supplementary Figure
Immune response of adult offspring splenocytes from poly(I:C)-treated dams.
