# Supplementary material for: Gestational Exposure to a Viral Mimetic Poly(I:C) Results in Long-Lasting Changes in Mitochondrial Function by Leucocytes in the Adult Offspring
Source: Mediators Inflamm. 2013 Sep 19;2013:609602. doi: 10.1155/2013/609602 (PMC3793312; doi:10.1155/2013/609602)
Supplement: Supplementary file 1 — Supplementary Methods Detailed description of cell culture conditions, cytokine quantification, and statistical analysis used for the immune response-related data. Supplementary Figure Immune response of adult offspring splenocytes from poly(I:C)-treated dams. [file 609602.f1.docx]

**Supplementary information for “Gestational exposure to a viral mimetic poly (I:C) results in long-lasting changes in mitochondrial function by leucocytes in the adult offspring” by Giulivi *et al*.**

**Methods**

***Cell Stimulation*-** Cells (5 x 10^6^/ml), isolated as described in the main manuscript, were stimulated for 24 h in a complete RPMI 1640 (ATCC modification; Invitrogen, Carlsbad, CA; catalog number A10491) media containing 25 mM glucose, 1 mM pyruvate and 2 mM glutamine, supplemented with 10% low endotoxin, heat inactivated fetal bovine serum (Invitrogen), 100 IU/ml penicillin, and 100 IU/ml streptomycin (Sigma, St Louis, MO), 25 µg/ml gentamycin (Sigma), 50 µM 2-mercaptoethanol (Sigma) with media alone or 10 µg/ml concavalin A (ConA; Sigma). After 24 h, cell supernatants were collected and stored at -80°C.

***Cytokine Analysis*-** The quantification of IL-1β, IL-6, IL-10, IL-17, TNF-α in supernatants was determined using murine multiplexing bead immunoassays (Millipore, Billerica, MA). Samples were run per manufacturer specifications. Specifically, 25 µl of supernatant, obtained as indicated above, was incubated with antibody-coupled beads. After a series of washes, a biotinylated detection antibody was added to the beads, and the reaction mixture was detected by the addition of streptavidin–phycoerythrin. The bead sets were analyzed using a flow-based Luminex™ 100 suspension array system (Bio-Plex 200; Bio-Rad Laboratories, Inc.). Sample cytokine concentrations were calculated by using the Bio-Plex Manager software with a standard curve derived from the known reference cytokine concentrations supplied by the manufacturer. A five-parameter model was used to calculate final concentrations and values were expressed in pg/ml. The sensitivity of this assay allowed the detection of cytokine concentrations above the following levels (in pg/ml): IL-1β (2.7), IL-6 (1.7), IL-10 (4.0), IL-17 (0.4), and TNF-α (0.7). Concentrations obtained below the sensitivity limit of detection (LOD) were calculated as LOD/2 for statistical comparisons. Cytokines and chemokines were tested on samples that underwent only one cycle of freeze-thaw.

***Statistical Analyses*-** The Mann-Whitney test was used for analysis of immune response-related data. Statistical significance was accepted at *p* ≤ 0.05.


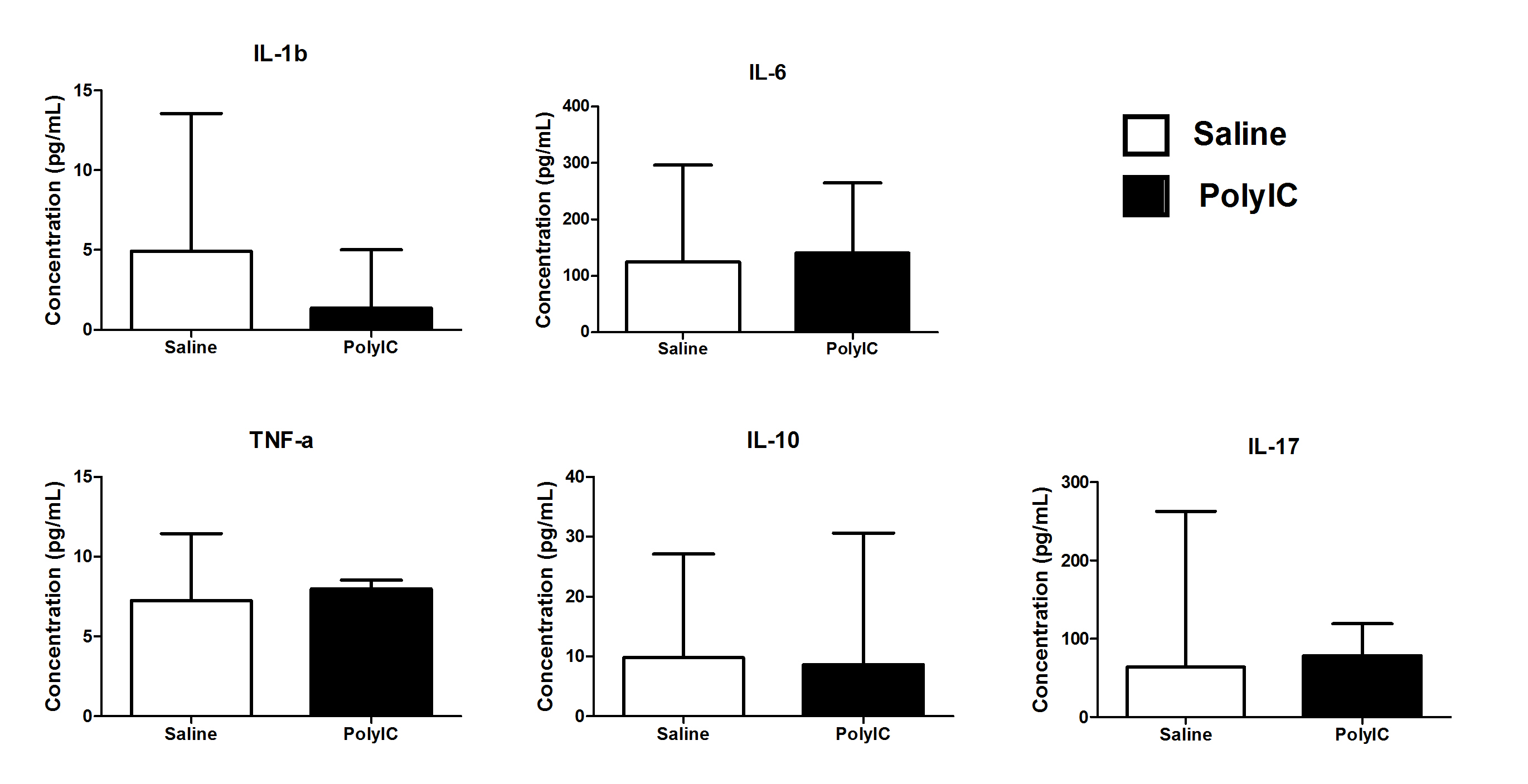


**Supplementary Figure 1**: **Immune response of adult offspring splenocytes.**

Splenocytes from offspring (10-12 weeks old) Poly(I:C) (*n* = 10) or saline (*n* = 10) treated dams at 12.5 gestational day were isolated and cultured in media for 24 h with 10 µg/ml concavalin, a T-cell agonist. Supernatants were analyzed by Luminex for cytokine production. Results are shown as median ± IQR.
